# Correlates of loneliness among university students

**DOI:** 10.1186/1753-2000-2-29

**Published:** 2008-10-13

**Authors:** Ugur Özdemir, Tarik Tuncay

**Affiliations:** 1 Faculty of Economics and Administrative Sciences Department of Social Work, Hacettepe University, Fatih Cd. No.195 PK.06290 Kecioren –, Ankara, Turkey

## Abstract

**Background:**

The purpose of this study was to investigate level of loneliness, essential needs during university education, and relationships between loneliness, essential needs, and characteristics of university students. A sample comprising 721 students participated in the study. The mean age was 21.58 (SD = 1.73) with a range from 18 to 25. The majority of the students were female (70.6%) and were living in students' dormitory (67.5%) with low (87.8%) income, away from their parents.

**Methods:**

The UCLA-R loneliness scale and sociodemographic questionnaire which includes an open-ended question on essential needs during university education were administered. Pearson-Product-Moment correlations were used to explore the relationships between participants' loneliness, needs, and characteristics.

**Results:**

It was found that 60.2% of the participants experienced loneliness. Economical support (81.6%), social interaction (46.9%) and psychosocial support (35%) were the essential needs during university education reported by the participants. The study findings indicate that there were significant relationships between the needs of economical support, social interaction, and loneliness level of university students. Results also show that there were significant relationships among romantic relationship, parents' status and loneliness. Participants' loneliness levels were relatively higher who had not any romantic relationship and were not from married families.

**Conclusion:**

The findings of this study provided essential information, about Turkish university students, concerning: level of loneliness and relationships that exist among loneliness, needs and sociodemographic characteristics. The findings also suggest implications for psychosocial practice. Because of the mean of loneliness were found to be high (45.49 ± 10.07), for this study, professionals need to pay attention to Turkish university students' psychosocial state, and need to empower them in establishing social relations.

## Background

Loneliness is a universal emotional and psychological experience. Loneliness is also seen as a normal experience that leads individual to achieve deeper self-awareness, a time to be creative, and an opportunity to attain self-fulfilment and to explore meaning of life [1,2]. Loneliness is also a condition of human life, an experience of humanizing which enables the person to sustain, extend, and deepen his/her humanity [3]. According to Weiss [4], loneliness is caused not by being alone but being without some definite needed relationship or set of relationships. Loneliness appears always to be a response to the absence of some particular relational provision, such as deficits in the relational provisions involved in social support [5].

However, the experience of loneliness is likewise unpleasant and distressing. Loneliness may also lead to people to submerge themselves into dependency relations, following direction, imitation, being like others, and striving for power and status [6,7]. Reading, watching TV, using the internet, social activities, attending parties, drinking, and also using drugs do not only signal loneliness, but these also may be some adaptive or maladaptive coping strategies university students use to overcome this unpleasant and distressing experience – loneliness [8]. Researchers have indicated that adolescents experience more loneliness than any other age groups [9]. Late adolescence and early adulthood (i.e., university age) are especially high risk for experiencing loneliness [10-12]. University is a transition period from being an adolescent to being an adult. It is a period for university students to seek and fulfil their sense of individuality and, at the same time, to seek and build close and social relationships with others. For many university students, this may be the first time they live away from their parents. They may move from the emotional and social support of their families. They leave home as well as their hometown friends. The separation of university students from their homes for the first time may create feelings of doubt, confusion, and anxiety, which the close companionship of residential halls may not totally prevent [4]. Once entering the university, they need to re-evaluate their past relationships with parents, teachers, friends, and girlfriend/boyfriend. They begin to learn how to deal with the attachment and separation processes of interpersonal situations in normal psychological growth and begin to create their own unique self-image. Lack of social and emotional support for university students, may lead to the experience of social and emotional loneliness [12].

For the most part, loneliness research has tended to focus on individual factors, that is, either on personality factors or lack of social contacts [5]. However, if one accepts the premise that loneliness is expressive of an individual's relationship to the community, then it is conceivable that the ways social relations are organized within the community will result in cross-cultural variations in the way people experience loneliness. Cross-cultural and individual differences, including personality [13], gender [2,14], and religious engagement [15] also have been considered as relevant factors in the study of loneliness.

Unfortunately, cross-cultural data about loneliness are scarce. The degree, frequency, and quality of a person's loneliness will be a function, among other things, of the society in which he or she lives [16]. In light of the growing awareness that research conducted in Western cultures does not necessarily represent the psychology of non-Western populations [17]. In this study authors examined the level of loneliness in Eurasian country, Turkey.

Because of the lack of psychosocial support and counselling services for students in Turkish universities [18,19] the psychological state and loneliness level of university students are unclear and need to be explored. To address this gap of knowledge, the objectives of this research were to identify and examine in Turkish university students: (1) level of loneliness, (2) characteristics and needs, and (3) correlations between loneliness, needs, and characteristics.

## Methods

### Participants and procedures

In this study, the sample set of the research was taken from three universities of Ankara by the random set sampling method. Using random sampling is the best way of ensuring that the observations are independent [20] and in this model, a researcher develops an accurate sampling frame according to a mathematically random procedure, then locates the exact element that was selected for inclusion in the sample [21]. All data were collected by two of the researchers between September and December 2007.

Each participant was informed, prior to the interview, about the purpose of the study, written informed consent was obtained, participants were told that they had the right to refuse participation and could withdraw at any time, and no inducements were offered to the sample for their participation. The participants completed the study via one-to-one interview in classrooms and participation was voluntary.

Socio-demographic characteristics of the participants are presented in Table 1 (*n *= 721). The mean age of the participants was 21.58 (SD = 1.73), with a range from 18 to 25 years. The majority of the participants were female (70.6%) and living in students dormitory (67.5%). and they had low and very low monthly income (87.8%). In Ankara, there are more women than men attending university education, and the sample reflects the university population on gender. 7.4% of the participants' parents were divorced and 59.6% of them had not a romantic relationship.

**Table 1:** Characteristics of the participants (n = 721)

**Variables**	**n (%)**
*Gender*	
Female	509 (70.6)
Male	212 (29.4)
*Age (years) mean (SD); range*	*21.58 (1.73); 18–25*
*Class*	
First year	134 (18.6)
Second year	340 (47.2)
Third year	139 (19.3)
Fourth year	108 (15.0)
*Monthly income (New Turkish Lira-YTL)*	
Up to 500 (very low)	283(39.3)
501–1000 (low)	350 (48.5)
1001–1500 (good)	57 (7.9)
Over 1501 (very good)	31 (4.3)
*Parents' Status*	
Divorced	53 (7.4)
Married	668 (92.6)
*Place of settlement*	
With family	152 (21.1)
Dormitory	487 (67.5)
At flat with friends	82 (11.4)
*Romantic Relationship*	
Yes	291 (40.4)
No	430 (59.6)

The distribution of class was: (1) first year, 18.6%, (2) second year, 47.2%, (3) third year, 19.3%, and (4) fourth year, 15.0%.

### Instruments

The study utilized the UCLA-R (University of California Los Angeles Loneliness Scale) Loneliness Scale to assess participants' degree of loneliness. The UCLA Loneliness Scale is one of the most widely used instruments to measure the subjective experience of loneliness. It has been used with varied populations, including the elderly [22], adolescents [16,18,23], college and university students [19,24]. The scale is a self-report measure, consisting of 20 items with 10 negatively stated (lonely) and 10 positively stated (non-lonely) items [25]. Participants were asked to respond to each item statement with responses of never, rarely, sometimes, and always. Higher scores on the loneliness scale indicate higher loneliness. The total scale mean scores, on the UCLA Loneliness Scale, among western students and students in Turkey normatively range from 36.56 to 40.08 [25,26].

As for the interpretation of the results, the number of participants scoring at least one standard deviation or more from the mean on UCLA-R Loneliness Scale was calculated [25,27]. Results indicated that 60.2% (n = 434) of the sample were "clinically" lonely.

The validity and reliability of this scale for Turkish society were studied by Demir, and the scale was found to have a high internal consistency (coefficient alpha .96 and high test-retest reliability of .94) [28]. In the present study, the Loneliness Scale had a Cronbach's Alpha of .82.

The researchers developed a questionnaire including, question on essential needs of students during university education and socio-demographic variables. The answer format of the question on participants' essential needs was open-ended. The participants were asked to write first three of their essential needs that take priority during their education. The answers were categorized by researchers into main themes such as economical support or social interaction etc.

The socio-demographic variables of questionnaire were as follows; gender (1 = female; 2 = male), age, class, monthly income, parents' status (1 = divorced; 2 = married), place of settlement, and romantic relationship. These variables were implemented as control variables.

### Statistical analyses

The analyses were conducted using the SPSS software version 14.0. Statistical analyses included descriptive statistics, reliability testing, and Pearson product moment correlation among variables. In descriptive statistics, proportion is used to describe categorical and numerical variables; mean and SD are used to describe continuous variables. Post hoc tests were not performed for socio-demographic variables and loneliness levels because there were fewer than three groups. Pearson product moment correlations were used to explore the relationships between loneliness, essential needs, and sociodemographic characteristics of university students. Levels of significance are indicated at both .05 and .01 in the correlation table.

## Results

The mean and standard deviation of loneliness was 45.49 ± 10.07 (Table 2). 60.2% (n = 434) of the participants exceeded a loneliness cut-off score of 46.49. The total loneliness score was found to be relatively high. The participants were asked to identify their essential needs during university education. Results were as follows; (1) economical support (%81.6), (2) social interaction (%46.9), (3) psychosocial support (%35.0), and (4) cultural activities (%8.3). See Table 2.

**Table 2:** Mean and standard deviation of loneliness and the needs of the participants (n = 721)

**Variables**	**M (SD)**	**Range**
*Loneliness*	45.49 (10.07)	20–74

*Needs*	**N**	%

Economical support	588	81.6
Social interaction	338	46.9
Psychosocial support	252	35.0
Cultural activities	60	8.3

The correlation coefficients among characteristics, loneliness and the needs of the participants are presented in Table 3. All tests were two-tailed and conducted at 5% significance. Class was found to be significantly negatively correlated with loneliness. The actual means and SDs for the UCLA-R among the different class ranks were as follows; the first year students 49.22 ± 8.73, the second year students 46.99 ± 8.35, the third year students 41.40 ± 11.27, and the fourth year students 41.50 ± 11.77. First year students, in particular, experience more loneliness than other university students. Researchers have indicated that the experience of loneliness during university life varies with time. Loneliness rose significantly during university entry and declined at the end of the fall semester [12]. See Table 3.

**Table 3:** Correlations among characteristics, loneliness and needs (n = 721)

**Variables**	**1**	**2**	**3**	**4**	**5**	**6**	**7**	**8**	**9**	**10**	**11**	**12**
*Characteristics*												
**1**. Gender	1	.23**	-.09*	-.01	.05	.05	.01	.03	-.03*	-.07*	-.15**	.10**
**2**. Age		1	.36**	.14**	.06	-.03	.03	.05	-.06	.01	-.17**	.08*
**3**. Class			1	.24**	.14**	-.22**	-.08*	-.28**	-.16**	-.05	-.14**	.03
**4**. Monthly income				1	.06	.00	.05	-.04**	.08*	-.14**	-.22**	-.04
**5**. Parents' Status			1		1	-.09**	-.06	-.31**	-.12**	-.00	-.05	.03
**6**. Place of settlement						1	-.41**	.61**	.01	-.03	-.00	-.05
**7**. Romantic relationship							1	.52**	.10**	.03	.04*	.00
**8**. *Loneliness*								1	.08*	-.02	.04**	.06

*Needs*												
**9**. Economical support									1	.13**	.11**	.05
**10**. Psychosocial support										1	.53**	.20**
**11**. Social interaction											1	.21**
**12**. Cultural activities												1

*p < .05; **p < .01 (two-tailed).

Gender was significantly negatively correlated with economical support, psychosocial support and social interaction, and significantly positively correlated with cultural activities indicating that female students in the present study had less expected economical and psychosocial support, and more expected cultural activities than male students. Age was negatively correlated with social interaction and positively correlated with cultural activities, indicating that older students had needed more cultural activities and less expected social relationships than younger students.

Not surprisingly, loneliness was significantly negatively correlated with parents' status, indicating that students from divorced families experience more loneliness than those from married families. Loneliness was also found to be significantly correlated with romantic relationship. This result indicates that loneliness levels of students who do not have a romantic relationship were found to be significantly higher than others.

The relationship between the needs of economical support, social interaction and loneliness showed a significant positive correlation. We found that participants, who reported the needs of economical support and social interaction, also had higher loneliness. Psychosocial support was significantly positively correlated with social interaction, and social interaction was also significantly positively correlated with cultural activities, indicating that these essential needs of participants are interrelated issues in the context of wellbeing of a university student.

## Discussion

The findings presented are tentative because a convenience sample was utilized in this study. They also should be interpreted cautiously since the basic design of the investigation consisted of cross-sectional sampling of the population.

In the present study, it was found that 60.2% of the Turkish university students from universities of Ankara were lonely, as evidenced by a mean loneliness score of 45.49. This finding is in agreement with those of a number of other studies in which it was found that cultural background plays a definitive role in the experience of loneliness [7,29-31]. As was shown in the literature, Western culture is characterized by individualism and independence. The result of this is that youngsters in Western countries are exposed to varying levels of autonomy, and are thus taught to become independent and self supportive. It could thus be that the Turkish university students are vulnerable to the experience of loneliness, because psychosocial support systems, of which the parental home is the foremost, may have been removed at an earlier stage, causing them to be more depressed and despairing. The primary reason given for this is that the Turkish social structure favours interdependence above autonomy. The present study strengthened this finding that many of the participants (67.5%) were living in students' dormitory, away from their parents.

There was no statistically significant correlation between the gender and age of the students and the level of loneliness. This finding was consistent with the results of many studies related to loneliness in adolescents and youngsters [18,32]. Although the amount of loneliness experienced by youngsters is not necessarily associated with any one sociodemographic characteristics, such as gender, age, or religion [23], a combination of such factors may be associated with loneliness, and the individual's developmental period may be cited as an important factor.

The researchers of this study also investigated the importance of economic status in the experience of loneliness. This variable was investigated because poverty was seen as an important determinant of loneliness [6,33]. The significant correlations were found among monthly income, need of economical support and loneliness of the participants. Monthly income was found to be negatively correlated with loneliness indicating that students who reported greater loneliness had lower income. Not surprisingly, need of economical support was also significantly correlated with loneliness. These findings will make a contribution to the cross-sectional research concerning the relationship between poverty and loneliness.

Research findings show that the loneliness levels of university students differentiate with respect to the existence of a romantic relationship. The loneliness levels of university students were found to be significantly correlated with romantic relationship. Weiss [4] stated that individuals who are unable to attach to other individuals will feel themselves lonely. The finding of this research given above confirms this statement of Weiss. This finding is also similar to the findings of Buyuksahin [10,34] who stated that loneliness of individuals who have close relationships is lower than the loneliness of other individuals.

The students from divorced families were found to be lonelier than those from intact. This finding is consistent with prior finding. Buyuksahin [34] found that Turkish students from married families experience less loneliness than those from divorced families. However, this may be the result of the inadequate interaction between parent and his/her child instead of the issue of divorce or single-parent family. Hojat [35] found that students who reported that their parents had not devoted enough time to them, or that their parents had never understood them, or that they had not gone to the parents for help, were more likely to experience loneliness.

The loneliness levels and needs of Turkish university students in Ankara were unclear. This research addressed this gap of knowledge in a large sample. This is the strength of our study. However, a potential limitation of the present study may be the results, although based on a large sample can only be generalized to other university students in a metropolis, Ankara. In developed countries, significant differences are not expected between different districts with regard to people's lifestyles – whereas in Turkey, which is a developing country and where rapid social changes are occurring, remarkable differences might be observed between different districts, especially between large and small cities [31,36].

## Conclusion

The findings of this study provided essential information, about Turkish university students, concerning: (1) level of loneliness, (2) characteristics and needs, and (3) relationships that exist among loneliness, needs and sociodemographic characteristics. The findings also suggest implications for psychosocial practice. Because of the mean of loneliness were high, for this study, professionals need to pay attention to university students' psychosocial state.

Because of the non-experimental and non-controlled design of this study, the generalizability of results may be limited. This study used a cross-sectional design, which investigates the real world at one point in time. Such a design does not examine longitudinal fluctuations in loneliness. Thus, longitudinal research is needed to examine psychosocial factors among university students. In addition, further study is needed to investigate psychosocial interventions that decrease loneliness level and facilitate adaptation and socialization among university students.

## Competing interests

The authors declare that they have no competing interests.

## Authors' contributions

UÖ conceived and planned the study and supervised data collection, TT and UÖ did literature search, TT analyzed the data and wrote the manuscript. All authors read and approved the manuscript.
